# The prevalence and correlates of burnout among Chinese preschool teachers

**DOI:** 10.1186/s12889-020-8287-7

**Published:** 2020-02-03

**Authors:** Shen Li, Yibo Li, Hao Lv, Rui Jiang, Peng Zhao, Xin Zheng, Lili Wang, Jie Li, Fuqiang Mao

**Affiliations:** 1 Department of Psychiatry, College of Basic Medical Sciences, Tianjin Medical University, 22 Qixiangtai Rd., Heping District, Tianjin, 300070 China; 2 Institute of Mental Health, Tianjin Anding Hospital, Tianjin Medical University, 13 Liulin Rd., Hexi District, Tianjin, 300222 China; 3 Institute of Psychology, Tianjin Medical University, 22 Qixiangtai Rd., Heping District, Tianjin, 300070 China

**Keywords:** Burnout, Stress, Preschool teachers, Depression

## Abstract

**Background:**

A series of studies have suggested that teachers are likely to experience professional burnout in various regions around the world. To date, no known research has been conducted to investigate the prevalence and correlates of burnout among preschool teachers in China. This study examined the level of self-reported burnout and correlates of burnout among Chinese preschool teachers.

**Methods:**

A cross-sectional study was conducted among1795 preschool teachers in Tianjin, China, during August 2018–October 2018. The validated Chinese version of the 15-item Maslach Burnout Inventory was used to assess burnout. A self-administered questionnaire collected the sociodemographic factors. The psychological factors were collected by the Chinese version of the 20-item Center for Epidemiologic Studies Depression Scale (CES-D) and the Perceived Stress Scale-14.

**Results:**

The prevalence of burnout in Chinese preschool teachers was 53.2% (95% CI:51%─56%). Burnout rate was significantly decreased in overweight (*P* = 0.001, OR = 0.58, 95% CI: 0.42–0.79) and obesity (*P* = 0.048, OR = 0.75, 95% CI: 0.56–1.00) teachers compared with teachers with normal weight. The type of school (*P* = 0.007, OR = 1.45, 95% CI: 1.11–1.91), income satisfaction (*P* = 0.001, OR = 0.67, 95% CI: 0.53–0.86), depression (*P* < 0.001, OR = 3.08, 95% CI: 2.34–4.05) and perceived stress (*P* < 0.001, OR = 1.15, 95%CI: 1.13–1.18) were significantly associated with burnout.

**Conclusions:**

The prevalence of burnout among preschool teachers in Tianjin, China, is high. Burnout was significantly associated with BMI, the type of school, income satisfaction, depression and perceived stress among Chinese preschool teachers.

## Background

Burnout is commonly defined as a psychological syndrome that results from exposure to a demanding work environment coupled with insufficient resources [1]. Maslach characterized in detail that burnout is a three-dimensional syndrome, including emotional exhaustion, depersonalization, and diminished professional accomplishment, which is resulted from workplace stressors [2].

Teaching stress is a prevalent and well-confirmed problem among teachers at various educational levels over recent decades. A series of studies have suggested that many teachers experience professional burnout and job dissatisfaction in various regions of the world [3–7]. However, the results of the prevalence of burnout in teachers are various.

In relation to the nature of a teacher’s job and to the context in which the professional work, burnout among teachers can affect teaching goals and educational environment, which may contribute to severe problems. Negative consequences associated with teacher burnout include poor job performance, health issues, and adverse student outcomes. Burnout was associated with job withdrawal-absenteeism, turnover intention, and actual attrition. However, for people who still work in school, burnout contributes to lower effectiveness and productivity at work [2]. Burnout was linked to job-related neurasthenia [2], depression [8] and voice disorder [9]. A study found that teachers’ status of burnout as an essential environmental factor is associated with students’ autonomous motivation [10]. In addition, burnout of preschool teachers hurt teacher-child interaction [11].

Researchers have attempted to investigate factors associated with burnout syndrome [9]. Correlates of burnout among teachers include some organizational factors, such as adverse working conditions [6],work stress [12, 13], lack of social support [14], interpersonal conflict [4], school type [15], administrative bureaucracy [5] and low salaries [5]. Meanwhile, several personal associations of burnout among teachers have also been indicated, such as age, education, marital status [16], emotional competence [17], temperament traits (emotional reactivity and perseveration), personality trait (extroversion, high neuroticism) [18, 19], motives for selecting a teaching profession [20], approval of a position as teacher [21] and self efficacy [22].

In China, no known research has been conducted to investigate the prevalence and correlates of burnout in preschool teachers. Building on the available data, this present study was performed with the aim of exploring the level of self-reported burnout and some correlates of burnout among Chinese preschool teachers.

## Methods

### Subjects

This cross-sectional survey was conducted in Tianjin, China, during July 2018–October 2018. Tianjin is a city with a population of more than 14 million inhabitants located in North China. There were 6 districts in urban areas with 197 kindergartens and 10 districts in suburbs with more than 1000 kindergartens including governmental and private types. One thousand seven hundred ninety-five preschool teachers randomly selected from 16 districts in Tianjin were invited to participate in the present survey. A power analysis based on a medium effect size was conducted in order to estimate necessary sample size.

The study population included governmental and private preschool teachers of both sexes. A complete medical history was obtained from all participants. All subjects were in good physical health and had no history of brain injury, mental system disease, cardiovascular disease, cancer, diabetes, thyroid dysfunction and chronic respiratory disease. No subjects were under the situations of any medical issues or disability after screening.

All participants in the current study were voluntary and were assured of the confidentiality and anonymity of the survey. The study protocol was approved by the Ethical Committee of Tianjin Anding Hospital and was conducted in accordance with the Declaration of Helsinki.

### Assessments tools

#### Assessment of socio-demographic factors

A self-administered questionnaire was designed to collect socio-demographic information. The following socio-demographic factors were assessed: sex (male/female), age, years of teaching, income satisfaction (yes/no), education background (senior high school or less, junior college, college or above), marital status (never married, married, divorced or widowed), the type of school (private school, public school) and body mass index (BMI, weight in kg /square of height in meters). BMI < 18.5, 18.5 ≤ BMI < 24, 24 ≤ BMI < 28 and BMI ≥ 28 were defined as underweight, normal weight, overweight and obese, respectively, in China [23, 24].

#### Assessment of burnout

All of the objects were measured with a Chinese version of the Maslach Burnout Inventory-General Survey scale (MBI-GS) [25, 26]. In the MBI-GS scale, burnout was assessed in three dimensions: emotional exhaustion (EE) (tiredness, somatic symptoms and decreased emotional resources), depersonalization (DP) (developing negative, cynical attitudes and impersonal feelings), and diminished professional accomplishment (PA) (feelings of incompetence, inefficiency and inadequacy) [27].

This is a self-report questionnaire with 15 items, rating on a 7-point scale (0 = never to 6 = each day). Total scores on each of the three subscales were stratified into high, moderate or low tertiles. The cutoffs for each tertile of burnout were empirically determined based on the previous data in the Chinese population: low EE < 11, moderate EE 11–15, high EE ≥ 15; low DP < 9, moderate DP 9–12, high DP ≥ 12;low PA < 19, moderate PA 19–22, high PA ≥ 22 [26]. The MBI has been translated into Chinese version and showed satisfactory reliability and validity; the internal consistency coefficient of the three dimensions was 0.896, 0.747, and 0.825 [28].

A score in the highest tertile for EE and DP and the lowest tertile for PA corresponds to a high level of burnout; we defined the outcome of “burnout” as reporting a high level of burnout on one or more subscales, i.e., in the highest tertile of EE or DP or the lowest tertile of PA [29].

#### Assessment of depression

Depression was measured by the Chinese version of the Center for Epidemiologic Studies Depression Scale (CES-D). This questionnaire consists of 20 items related to characteristic symptoms and depressive behaviors, with each item rated from 0 to 3. The CES-D has been widely used in Chinese populations with excellent reliability and validity [30]. The total score ranges from 0 to 60, and CES-D ≥ 16 indicates that respondents may be more likely to be depressed [31].

#### Assessment of perceived stress

The perceived stress levels of participants were measured by the Perceived Stress Scale-14 Chinese version (PSS-14) [32, 33]. The PSS-14 is widely used by psychologists to assess perceived stress levels. This is a self-report questionnaire with 14 items, rating on a 5-point scale (0 = never to 4 = always).

### Data analysis

All analyses were done by using IBM SPSS (version 19.0). Frequencies and percentages were summarized for the categorical variables. Mean and standard deviation (SD) were calculated for continuous numerical data. Comparisons of sociodemographic and psychological variables between different groups were analyzed by using the chi-square test for categorical variables or independent samples *t*-tests for continuous variables.

For the examination of correlates of burnout, a two-step procedure was followed. Firstly, a univariate logistic regression analysis was done to identify potential associations of burnout. Secondly, a multivariable analysis using binary logistic regression was conducted to determine the relative predictors of burnout when controlled for potential confounding among the various predictor variables. Correlates with a *P*-value < 0.1 in the bivariate analysis were included in the multivariable analysis using the “Enter” method. We reported the crude odds ratios (OR) with 95% confidence intervals (CI) in univariate analysis and adjusted odds ratios (AOR) with 95% CI in the multivariable analysis. All *P*-values were two-tailed with a statistically significant level at 0.05.

## Results

### Demographic and psychological characteristics of Chinese preschool teachers

The response rate was 97.0% (1741/1795). A total of 1741 Chinese preschool teachers, including 134 male and 1607 female, were included in our study. The average age of teachers was 34.66 [SD = 8.86], ranging from 18 to 48 years. The average years of teaching were 13.54 [SD = 10.99], ranging from 1 to 35. Of them, 53.9% had a bachelor degree or above, 29.7% junior college degree, 16.4% high school or less. The majority (75.4%) were married. The majority (67.3%) were from public school. The minority (38.1%) were satisfied with their income. Of them, the prevalence of underweight, overweight, and obesity was 7.5, 19.8, and 25.5%, respectively. The percentage of depression was 39.9%. The average scores of perceived stress were 23.98 [SD = 8.85], ranging from 0 to 56 (Table 1).

**Table 1:** Characteristics of Chinese preschool teachers with or without burnout

Variables	All teachers	Teachers without burnout	Teachers with burnout	*t* or *χ*^*2*^	*P* value
	(*N* = 1741)	(*N* = 815)	(*N* = 926)		
Sex				0.45	0.502
Male	134/1741(7.7%)	59/815(7.2%)	75/926(8.1%)		
Female	1607/1741(92.3%)	756/815(92.8%)	851/926(91.9%)		
Education				3.64	0.162
Senior high school or less	266/1741(16.4%)	122/815(15.0%)	164/926(17.7%)		
Junior college	516/1741(29.7%)	256/815(31.4%)	260/926(28.1%)		
College or above	939/1741(53.9%)	437/815(53.6%)	502/926(54.2%)		
Marital status				14.09	**0.001**
Never married	361/1741(20.7%)	139/815(17.1%)	222/926(24.0%)		
Married	1313/1741(75.4%)	648/815(79.5%)	665/926(71.8%)		
Divorced or Widowed	67/1741(3.9%)	28/815(3.4%)	39/926(4.2%)		
The type of school				2.74	0.098
Private school	570/1741(32.7%)	283/815(34.7%)	287/926(31.0%)		
Public school	1171/1741(67.3%)	532/815(65.3%)	639/926(69.0%)		
Income satisfaction				49.52	**< 0.001**
No	1077/1741(61.9%)	433/815(53.1%)	644/926(69.5%)		
Yes	664/1741(38.1%)	382/815(46.9%)	282/926(30.5%)		
BMI				18.04	**< 0.001**
Normal weight	822/1741(47.2%)	371/815(45.5%)	451/926(48.7%)		
Underweight	130/1741(7.5%)	44/815(5.4%)	86/926(9.3%)		
Overweight	345/1741(19.8%)	188/815(23.1%)	157/926(17.0%)		
Obesity	444/1741(25.5%)	212/815(26.0%)	232/926(25.0%)		
Depression				359.56	**< 0.001**
No	1046/1741(60.1%)	683/815(83.8%)	363/926(39.2%)		
Yes	695/1741(39.9%)	132/815(16.2%)	563/926(60.8%)		
Age	34.66 ± 8.86	35.53 ± 8.83	33.89 ± 8.82	3.87	**< 0.001**
Years of teaching	13.54 ± 10.99	14.37 ± 11.24	12.81 ± 10.72	2.95	**0.003**
Perceived stress	23.98 ± 8.85	19.05 ± 7.61	28.32 ± 7.49	−25.56	**< 0.001**

Boldface indicates significant at *P* < 0.01

*BMI* body mass index

### Prevalence of burnout in Chinese preschool teachers

**Fig. 1:**
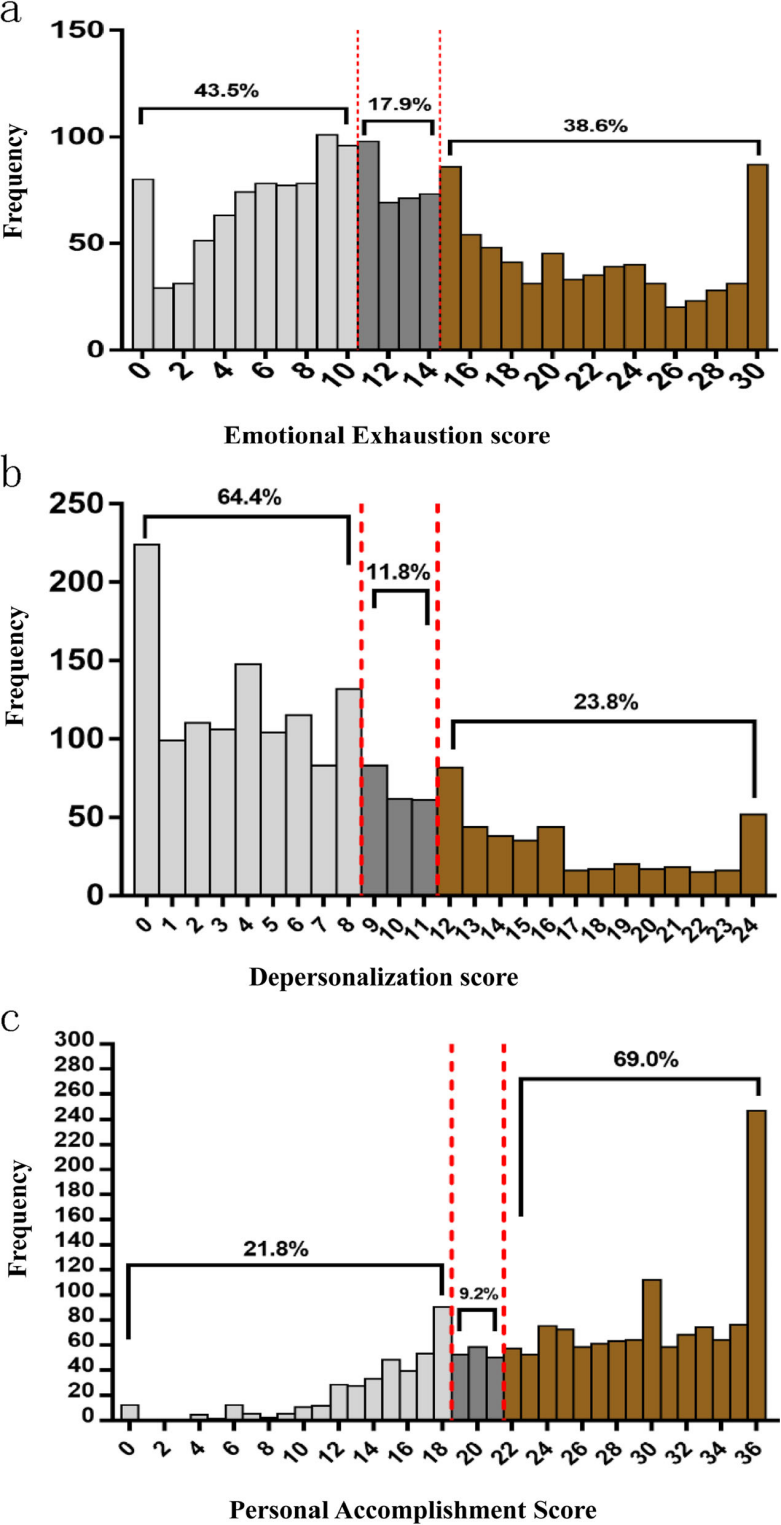
Results of the Maslach Burnout Inventory-General Survey (MBI-GS) Subscale Scores. Histograms a to c represented the score distribution of the MBI-GS Subscale of Emotional Exhaustion (EE), Depersonalization (DP), and Personal Accomplishment (PA), respectively. Vertical axis in histogram **a** demonstrated the frequency of scores for EE; histogram **b** is for DP; and histogram **c** is for PA. Each graph was divided into three parts from left to right, which respectively represent low, medium and high scores

Respectively, 38.6%(673/1741) and 23.8% (415/1741) of preschool teachers reported a high level of emotional exhaustion and depersonalization, while 21.8% (382/1741) showed low levels of professional accomplishment (Fig. 1). The prevalence of burnout in Chinese preschool teachers was 53.2% (928/1741, 95% CI = 51–56%). The prevalence estimate of burnout was 53.0% (851/1607) in female subjects. The prevalence estimate of burnout was 56.0% (75/134) in male subjects.

Additional file 1: Table S1 summarizes the mean total scores and the mean item scores of the three subscales of the MBI-GS among Chinese preschool teachers. The mean total scores (SD) were 13.18 (8.28) for EE, 7.50 (6.30) for DP, and 25.82 (8.01) for PA in our study. The mean item scores (SD) were 2.64 (1.66) for EE, 1.87 (1.58) for DP, 4.30 (1.33) for PA in our study.

### Comparison of demographic and psychological variables between non-burnout and burnout group

Profiles of demographic and psychological variables of Chinese preschool teachers are shown in Table 1. The teachers with burnout have significantly higher perceived stress levels (*P* < 0.001), shorter years of teaching (*P* < 0.01) as compared to the teachers without burnout. The teachers with burnout are younger than the teachers without burnout (*P* < 0.001). There are significant differences in marital status, income satisfaction, BMI, and depression status between non-burnout and burnout group (all *P* < 0.001). However, there is no significant difference in sex, the type of school, the educational background between non-burnout and burnout group (all *P* > 0.05).

### Sex differences in comparison of demographic and psychological variables between non-burnout and burnout group

#### Comparison of demographic and psychological variables between non-burnout and burnout group in women

The teachers with burnout have significantly younger age (*P* < 0.01), higher perceived stress levels (*P* < 0.001), shorter years of teaching (*P* < 0.05) as compared to the teachers without burnout. There are significant differences in marital status, income satisfaction, BMI, and depression status between non-burnout and burnout group (all *P* < 0.05). However, there is no significant difference in the type of school and the educational background between non-burnout and burnout group (all *P* > 0.05). (Additional file 1: Table S2).

#### Comparison of demographic and psychological variables between non-burnout and burnout group in men

The teachers with burnout have significantly younger age (*P* < 0.01), higher perceived stress levels (*P* < 0.001), shorter years of teaching (*P* < 0.05) as compared to the teachers without burnout. There are significant differences in marital status, income satisfaction, and depression status between non-burnout and burnout group (all *P* < 0.01). However, there is no significant difference in BMI, the type of school and the educational background between non-burnout and burnout group (all *P* > 0.05). (Additional file 1: Table S3).

#### Correlates of burnout

In the bivariate analysis, nine factors showed a *P*-value < 0.1 (Table 2). These included a series of sociodemographic and psychological factors. The nine predictors identified at bivariate analysis were included in the multivariable analysis. Table 2 summarizes the results of the multivariable analysis of the correlates of burnout. Multivariable analysis elicited five statistically significant associations with burnout when controlled for other factors included in the model. Income satisfaction showed a statistically significant negative association with burnout (*P* = 0.001, OR = 0.67, 95% CI: 0.53–0.86). The preschool teachers who worked in the public school had a statistically significant higher likelihood of having burnout in comparison to their counterparts (*P* = 0.007, OR = 1.45, 95% CI: 1.11–1.91). Compared with normal-weight subjects, overweight *(P* = 0.001, OR = 0.58, 95% CI: 0.42–0.79) and obesity (*P* = 0.048, OR = 0.75, 95% CI: 0.56–1.00) correlated with a lower likelihood of having burnout. Perceived stress showed statistically significant positive associations with burnout (*P* < 0.001, OR = 1.15, 95% CI: 1.13–1.18). The depressed preschool teachers were more likely to have burnout as opposed to their counterparts (*P* < 0.001, OR = 3.08, 95% CI: 2.34–4.05).

**Table 2:** Crude and independent correlates of burnout

Factor	Level	Univariate regression analysis				Multivariate factor regression analysis^a^			
		Wald *χ*^*2*^	OR	95%CI	*P value*	Wald *χ*^*2*^	OR	95%CI	*P value*
Sex	Male	–	1.00	–	–	–	–	–	–
	Female	0.45	0.89	0.62–1.26	0.502	–	–	–	–
Education		3.63			0.163	5.15			0.076
	Senior high school or less	–	1.00	–	–	–	1.00	–	–
	Junior college	3.57	0.76	0.57–1.01	0.059	3.68	0.70	0.49–1.01	0.055
	College or above	1.33	0.86	0.65–1.12	0.249	0.23	0.92	0.65–1.30	0.635
Marital status		13.99			**0.001**	0.94			0.624
	Never married	–	1.00	–	–	–	1.00	–	–
	Married	13.27	0.64	0.51–0.82	**< 0.001**	13.27	0.95	0.67–1.36	0.793
	Divorced or Widowed	0.26	0.87	0.51–1.48	0.613	0.26	0.71	0.35–1.45	0.343
The type of school	Private school	–	1.00	–	–	–	1.00	–	–
	Public school	2.74	1.18	0.97–1.45	0.098	2.74	1.45	1.11–1.91	**0.007**
Income satisfaction	No	–	1.00	–	–	–	1.00	–	–
	Yes	48.94	0.50	0.41–0.60	**< 0.001**	48.94	0.67	0.53–0.86	**0.001**
BMI		17.76			**0.001**	15.54			**0.001**
	Normal weight	–	1.00	–	–	–	1.00	–	–
	Underweight	5.48	0.63	0.43–0.93	**0.019**	5.48	1.28	0.79–2.06	0.318
	Overweight	15.71	0.43	0.28–0.65	**< 0.001**	15.71	0.58	0.42–0.79	**0.001**
	Obesity	7.75	0.56	0.37–0.84	**0.005**	7.75	0.75	0.56–1.00	**0.048**
Depression	No	–	1.00	–	–	–	1.00	–	–
	Yes	319.59	8.03	6.39–10.08	**< 0.001**	319.59	3.08	2.34–4.05	**< 0.001**
Age (years)		14.78	0.98	0.97–0.99	**< 0.001**	14.78	1.01	0.98–1.04	0.447
Years of teaching		8.66	0.99	0.98–1.00	**0.003**	8.66	1.01	0.99–1.03	0.550
Perceived stress		344.70	1.19	1.17–1.21	**< 0.001**	344.70	1.15	1.13–1.18	**< 0.001**

Boldface indicates significant at *P* < 0.05

*BMI* body mass index

^a^This multivariate factor regression analysis was based on n = 1741 and adjustment for all variables with the exception of sex

### Sex differences in correlates of burnout

#### Correlates of burnout in women

In the bivariate analysis, nine factors showed a *P*-value < 0.1 (Table 3). The nine predictors identified at bivariate analysis were included in the multivariable analysis. Table 3 summarizes the results of the multivariable analysis of the correlates of burnout in women. Multivariable analysis elicited six statistically significant associations with burnout when controlled for other factors included in the model. Compared with the subjects with college or above degree, the subjects with junior college degree are less likely to have burnout (*P* = 0.015, OR = 0.70, 95% CI: 0.53–0.93). Income satisfaction showed a statistically significant negative association with burnout (*P* = 0.006, OR = 0.70, 95% CI: 0.54–0.90). The preschool teachers who worked in the public school had a statistically significant higher likelihood of having burnout in comparison to their counterparts (*P* = 0.004, OR = 1.51, 95% CI: 1.14–2.01). Compared with normal-weight subjects, overweight *(P* = 0.001, OR = 0.55, 95% CI: 0.39–0.78) correlated with a lower likelihood of having burnout. Perceived stress showed statistically significant positive association with burnout (*P* < 0.001, OR = 1.16, 95% CI: 1.13–1.18). The depressed preschool teachers were more likely to have burnout as opposed to their counterparts (*P* < 0.001, OR = 3.22, 95% CI: 2.41–4.31).

**Table 3:** Crude and independent correlates of burnout in female subjects

Factor	Level	Univariate regression analysis				Multivariate factor regression analysis^a^			
		Wald *χ*^*2*^	OR	95%CI	*P value*	Wald *χ*^*2*^	OR	95%CI	*P value*
Education		3.92			0.141	6.94			**0.031**
	College or above	–	1.00	–	–	–	1.00	–	–
	Junior college	3.17	0.76	0.56–1.03	0.075	5.88	0.70	0.53–0.93	**0.015**
	Senior high school or less	0.47	0.91	0.68–1.20	0.494	0.02	1.03	0.71–1.50	0.888
Marital status		8.68			**0.013**	2.11			0.348
	Never married	–	1.00	–	–	–	1.00	–	–
	Married	8.33	0.70	0.54–0.89	**0.004**	0.02	1.02	0.70–1.50	0.901
	Divorced or Widowed	0.22	0.88	0.50–1.53	0.641	1.48	0.62	0.29–1.34	0.224
The type of school	Private school	–	1.00	–	–	–	1.00	–	–
	Public school	3.39	1.22	0.99–1.50	0.066	8.14	1.51	1.14–2.01	**0.004**
Income satisfaction	No	–	1.00	–	–	–	1.00	–	
	Yes	41.65	0.51	0.42–0.63	**< 0.001**	7.66	0.70	0.54–0.90	**0.006**
BMI		16.04			**0.001**	14.96			**0.002**
	Normal weight	–	1.00	–	–	–	1.00	–	–
	Underweight	4.52	1.53	1.03–2.27	**0.034**	0.87	1.26	0.78–2.05	0.350
	Overweight	8.53	0.67	0.51–0.88	**0.003**	11.72	0.55	0.39–0.78	**0.001**
	Obesity	0.56	10.9	0.72–1.16	0.453	3.03	0.77	0.57–1.03	0.082
Depression	No	–	1.00	–	–	–	1.00	–	–
	Yes	300.38	8.37	6.58–10.65	**< 0.001**	62.45	3.22	2.41–4.31	**< 0.001**
Age (years)		10.04	0.98	0.97–0.99	**0.002**	0.61	1.01	0.98–1.05	0.436
Years of teaching		5.52	0.99	0.98–1.00	**0.019**	0.25	1.01	0.98–1.03	0.616
Perceived stress		344.70	1.19	1.17–1.22	**< 0.001**	174.87	1.16	1.13–1.18	**< 0.001**

Boldface indicates significant at *P* < 0.05

*BMI* body mass index

^a^This multivariate factor regression analysis was based on *n* = 1607 and adjustment for all variables

#### Correlates of burnout in men

In the bivariate analysis, seven factors showed a *P*-value < 0.1 (Additional file 1: Table S4). The seven predictors identified at bivariate analysis were included in the multivariable analysis. Additional file 1: Table S4 summarizes the results of the multivariable analysis of the correlates of burnout in men. Multivariable analysis elicited two statistically significant associations with burnout when controlled for other factors included in the model. Income satisfaction showed a statistically significant negative association with burnout (*P* = 0.045, OR = 0.40, 95% CI: 0.16–0.98). Perceived stress showed statistically significant positive associations with burnout (*P* = 0.002, OR = 1.11, 95% CI: 1.04–1.19).

## Discussion

The present study, which firstly investigated burnout among preschool teachers in China, showed a high rate of burnout (53.2%) among the participants. It is higher than the reported values of the prevalence of burnout among teachers in some previous studies from different countries, e.g. 29% in Brazil, 24.5% in Israel, 21% in Venezuela [3, 7, 34], possibly due to occupational settings, sociocultural factors, different measures of assessment or different criterions for burnout. Results also showed that 56.5% of preschool teachers presented medium or high levels of emotional exhaustion, 35.6% a medium or high level of depersonalization, and 21.8% a low level of professional accomplishment. A similar prevalence of burnout in preschool teachers was reported in Italy (53.4% of preschool teachers presented medium or high levels of emotional exhaustion, 50% a medium or high level of depersonalization and 18.5% a low level of professional accomplishment) [4].

Our study also identified a series of correlates of burnout in preschool teachers in China. Sociodemographic factors, such as the type of school, income satisfactory and BMI, emerged as statistically significant predictors with burnout (Table 2). We found that teachers who worked in public schools showed a higher rate of burnout than teachers who worked in private schools, which is consistent with previous studies [15, 35]. There are higher job requirements, stricter teaching management and assessment in the public kindergartens than in the private kindergartens and parents have higher expectations for public kindergarten teachers, which maybe inevitably lead to more mental pressure on public kindergarten teachers. Previous studies have indicated that occupational stress was a risk factor for teachers burnout [17, 36]. On the other hand, teachers in public schools had statistically significant higher educational qualifications than their counterparts (*χ*^*2*^ = 214.14, *P* < 0.001). Therefore, it can be assumed that the teachers in public schools have higher career expectations so that they are more likely to experience a lower level of professional accomplishment when facing development difficulties.

This evaluation also demonstrated that the preschool teachers who were not satisfied with their income were at a greater risk of having burnout in comparison to their counterparts. Other studies found that low paid teachers were more likely to experience burnout [5, 16], similar to our results. It can be assumed that reduced income satisfactory would lead to more deficient working motivation, which contributes to burnout.

Interestingly, we found that overweight and obese teachers in our study had a substantially lower likelihood of having burnout comparing to the teachers whose BMI were healthy. Generally speaking, overweight and obesity were negatively correlated with worse outcomes [37–39]. However, several studies showed that excess body weight could decrease the morality of patients [40–42], which is called the “obesity paradox.” Some studies have indicated that the “obesity paradox” phenomenon also existed between BMI and health-related quality of life (HRQL) [43, 44]. The type I obese was significantly correlated with higher mental component summary scores of HRQL in comparison to the healthy weight [43]. A recent study showed that better HRQL predicted a lower likelihood of having burnout [45]. Therefore, overweight and obese may decrease the risk of burnout by promoted health-related quality of life. More researches were needed to explore the underlying mechanism.

Whether there is an association between burnout and major depressive disorder is currently an unresolved, active debate with compelling arguments on both sides [46]. Our study indicated a strong positive association of burnout with depression. Kallay et al. found that teachers who reported higher levels of emotional exhaustion or higher levels of depersonalization or lower levels of professional accomplishment also reported significantly higher levels of depression [47], consistent with our results. In a Korea study, Kindergarten school teachers’ burnout was influenced by depression [8]. One underlying mechanism by which depression and burnout may be associated is via individual personality traits such as neuroticism. Neurotic individuals tended to express higher stress reactions and more negative emotions, leading them more susceptible to both psychopathology and burnout [48].

In line with many studies [8, 14, 17, 49], we found that burnout was significantly positively related to perceived stress. The self-determination theory identified three basic psychological needs, including autonomy, competence, and relatedness. In a Spanish study [50], teachers’ basic psychological needs thwarting were predicted by perceived job pressure. At the same time, the thwarting of basic needs was significantly associated with burnout [50, 51]. Therefore, higher perceived stress in teachers may increase the risk of burnout by thwarted autonomy, competence, and relatedness needs. Besides, Rey et al. found that weak recovery partially mediated the relationship between work stress and reduced professional efficacy [12]. Therefore, poor healing may be one possible mechanism that can help to understand why perceived stress was associated with burnout. More researches were needed to explore the underlying mechanism.

In order to exploring the sex differences of the burnout in Chinese preschool teachers, we also analyzed the characteristics and independent correlates of burnout in female and male subjects independently. In the female preschool teachers, education is an independent correlate of burnout, suggesting more education maybe improve the female preschool teacher to deal with the burnout which needs further research. In the male preschool teachers, the correlates of burnout were much more different than the total participates and the female teachers. Income satisfaction and perceived stress are the independent correlate of burnout in the male preschool teachers. In China, the preschool teachers are mainly females, which may be due to the culture, the characteristics of this occupation and some factors related to the burnout. The smaller sample size of male preschool teachers in the multivariable analysis made the statistical test less powerful. Further researches are still needed to conduct an in-depth analysis in larger male samples.

There are some limitations related to this study. First, we cannot make inferences of causality among variables because of the nature of this study (cross-sectional assessment). Another limitation is the lack of detection of biomarker, such as stress-related proteins and genes which maybe help us to find some indicator about burnout. This study was limited to the district of Tianjin. However, the findings can be reasonably representative of the preschool teachers throughout China, since other kindergartens throughout China are similar to those in Tianjin, such as circumstances and kids. The considerable strengths of our study include the first research in a larger, representative sample about burnout among the Chinese preschool teachers who need more attention. The high prevalence of burnout and the correlations of burnout in this study are significant.

## Conclusion

Summarizing the findings in the conclusion, the prevalence of burnout among preschool teachers in China is high. Multivariable analysis elicited some statistically significant correlates, including sociodemographic factors and psychological factors. So it is necessary to take corresponding measures to prevent burnout among preschool teachers according to correlates of burnout.

## Supplementary informations

**Additional file 1**: **Table S1.** Descriptive statistics of MBI-GS subscale scores among Chinese preschool teachers (*N* = 1741). **Table S2.** Characteristics of female subjects with or without burnout. **Table S3.** Characteristics of male subjects with or without burnout. **Table S4.** Crude and independent correlates of burnout in male subjects.

## Data Availability

The datasets analyzed during the current study are not publicly available; and the materials used in the present study is available by request from all academic based researchers by a contact to the corresponding author.

## Abbreviations

AOR: Adjusted odds ratios
BMI: Body mass index
CES-D: Center for Epidemiologic Studies Depression Scale
CI: Confidence intervals
DP: Depersonalization
EE: Emotional exhaustion
HRQL: Health-related quality of life
MBI-GS: Maslach Burnout Inventory-General Survey scale
OR: Odds ratios
PA: Professional accomplishment
PSS-14: Perceived Stress Scale-14
SD: Standard deviation
