# Supplementary material for: The prevalence and correlates of burnout among Chinese preschool teachers
Source: BMC Public Health. 2020 Feb 3;20:160. doi: 10.1186/s12889-020-8287-7 (PMC6998270; doi:10.1186/s12889-020-8287-7)
Supplement: Supplementary file 1 — Additional file 1: Table S1. Descriptive statistics of MBI-GS subscale scores among Chinese preschool teachers (N = 1741). Table S2. Characteristics of female subjects with or without burnout. Table S3. Characteristics of male subjects with or without burnout. Table S4. Crude and independent correlates of burnout in male subjects. [file 12889_2020_8287_MOESM1_ESM.docx]

**Additional file**

**The prevalence and correlates of burnout among Chinese preschool teachers**

Shen Li^a,^^b*^, Yibo Li^a,c*^, Hao Lv^b*^, Rui Jiang^a,b^, Peng Zhao^a^, Xin Zheng^b^, Lili Wang^b^, Jie Li^b#^, Fuqiang Mao^a,c#^

**Table S1.** Descriptive statistics of MBI-GS subscale scores among Chinese preschool teachers (*N*=1741)

| Subscale | Mean total score | SD | Mean item score | SD |
| --- | --- | --- | --- | --- |
| EE | 13.18 | 8.28 | 2.64 | 1.66 |
| DP | 7.50 | 6.30 | 1.87 | 1.58 |
| PA | 25.82 | 8.01 | 4.30 | 1.33 |

Abbreviations: EE, emotional exhaustion; DP, depersonalization; PA, professional accomplishment; SD, standard deviation.

**Table S2.** Characteristics of female [subject](javascript:;)s with or without burnout

| Characteristics | All female teachers | Female teachers without burnout | Female teachers  with burnout | *t* or *χ^2^* | *P* value |
| --- | --- | --- | --- | --- | --- |
|  | (N=1607) | (N=756) | (N=851) |  |  |
| Education |  |  |  | 3.92 | 0.141 |
| College or above | 860/1607 (53.5%) | 396/756 (52.4%) | 464/851 (54.5%) |  |  |
| Junior college | 497/1607 (30.9%) | 251/756 (33.2%) | 246/851 (28.9%) |  |  |
| Senior high school or less | 250/1607 (15.6%) | 109/756 (14.4%) | 141/851 (16.6%) |  |  |
| Marital status |  |  |  | 8.72 | **0.013** |
| Never married | 329/1607 (20.5%) | 132/756 (17.5%) | 197/851 (23.1%) |  |  |
| Married | 1218/1607 (75.8%) | 598/756 (79.1%) | 620/851 (72.9%) |  |  |
| Divorced or Widowed | 60/1607 (3.7%) | 26/756 (3.4%) | 34/851 (4.0%) |  |  |
| The type of school |  |  |  | 3.39 | 0.066 |
| Private school | 537/1607 (33.4%) | 270/756 (35.7%) | 267/851 (31.4%) |  |  |
| Public school | 1070/1607 (66.6%) | 486/756 (64.3%) | 584/851 (68.6%) |  |  |
| Income satisfaction |  |  |  | 42.11 | **<0.001** |
| No | 999/1607 (62.2%) | 407/756 (53.8%) | 592/851 (69.6%) |  |  |
| Yes | 608/1607 (37.8%) | 349/756 (46.2%) | 259/851 (30.4%) |  |  |
| BMI |  |  |  | 16.25 | **0.001** |
| Normal weight | 775/1607 (48.2%) | 352/756 (46.5%) | 423/851 (49.7%) |  |  |
| Underweight | 125/1607 (7.8%) | 44/756 (5.8%) | 81/851 (9.5%) |  |  |
| Overweight | 294/1607 (18.3%) | 163/756 (21.6%) | 131/851 (15.4%) |  |  |
| Obesity | 413/1607 (25.7%) | 197/756 (26.1%) | 216/851 (25.4%) |  |  |
| Depression |  |  |  | 340.38 | **<0.001** |
| No | 975/1607 (60.7%) | 639/756 (84.5%) | 336/851 (39.5%) |  |  |
| Yes | 632/1607 (39.3%) | 117/756 (15.5%) | 515/851 (60.5%) |  |  |
| Age | 34.61±8.84 | 35.35±8.80 | 33.95±8.83 | 3.18 | **0.001** |
| Years of teaching | 13.42±10.96 | 14.10±11.12 | 12.81±10.79 | 2.35 | **0.019** |
| Perceived stress | 23.93±8.83 | 18.99±7.56 | 28.33±7.44 | -24.92 | **<0.001** |

Boldface indicates significant at *P*< 0.05; BMI: Body mass index.

**Table S3.** Characteristics of male [subject](javascript:;)s with or without burnout.

| Characteristics | All Male teachers | Male teachers without burnout | Male teachers  with burnout | *t* or *χ^2^* | *P* value |
| --- | --- | --- | --- | --- | --- |
|  | (N=134) | (N=59) | (N=75) |  |  |
| Education |  |  |  | 5.32 | 0.070 |
| College or above | 79/134 (59.0%) | 41/59 (69.5%) | 38/75 (50.7%) |  |  |
| Junior college | 19/134 (14.1%) | 5/59 (8.5%) | 14/75 (18.6%) |  |  |
| Senior high school or less | 36/134 (26.9%) | 13/59 (22.0%) | 23/75 (30.7%) |  |  |
| Marital status |  |  |  | 9.91 | **0.007** |
| Never married | 32/134 (23.9%) | 7/59 (11.9%) | 25/75 (33.3%) |  |  |
| Married | 95/134 (70.9%) | 50/59 (84.7%) | 45/75 (60.0%) |  |  |
| Divorced or Widowed | 7/134 (5.2%) | 2/59 (3.4%) | 5/75 (6.7%) |  |  |
| The type of school |  |  |  | 0.38 | 0.537 |
| Private school | 33/134 (24.6%) | 13/59 (22.0%) | 20/75 (26.7%) |  |  |
| Public school | 101/134 (75.4%) | 46/59 (78.0%) | 55/75 (73.3%) |  |  |
| Income satisfaction |  |  |  | 8.67 | **0.003** |
| No | 78/134 (58.2%) | 26/59 (44.1%) | 52/75 (69.3%) |  |  |
| Yes | 56/134 (41.8%) | 33/59 (55.9%) | 23/75 (30.7%) |  |  |
| BMI |  |  |  | 0.38 | 0.537 |
| Normal weight | 47/134 (35.1%) | 19/59 (32.2%) | 28/75 (37.3%) |  |  |
| Underweight/ Overweight  / Obesity | 87/134 (64.9%) | 40/59 (67.8%) | 47/75 (62.7%) |  |  |
| Depression |  |  |  | 19.73 | **<0.001** |
| No | 71/134 (53.0%) | 44/59 (74.6%) | 27/75 (53.0%) |  |  |
| Yes | 63/134 (47.0%) | 15/59 (25.4%) | 48/75 (47.0%) |  |  |
| Age | 35.24±9.13 | 37.81±8.96 | 33.21±8.81 | 2.98 | **0.003** |
| Years of teaching | 15.05±11.27 | 17.85±12.29 | 12.85±9.94 | 2.60 | **0.010** |
| Perceived stress | 24.55±9.15 | 19.86±8.23 | 28.24±8.13 | -5.89 | **<0.001** |

Boldface indicates significant at *P*< 0.05; BMI: body mass index.

**Table S4.** Crude and independent correlates of burnout in male [subject](javascript:;)s.

| Factor | Level | Univariate regression analysis | | | | Multivariate factor regression analysis**^#^** | | | |
| --- | --- | --- | --- | --- | --- | --- | --- | --- | --- |
|  |  | Wald *χ^2^* | OR | 95%CI | *P value* | Wald *χ^2^* | OR | 95%CI | *P value* |
| Education |  | 5.16 |  |  | 0.076 | 3.61 |  |  | 0.164 |
|  | Senior high school or less | - | 1.00 | - | - | - | 1.00 | - | - |
|  | Junior college | 0.54 | 1.58 | 0.46-5.40 | 0.463 | 1.57 | 2.58 | 0.59-11.32 | 0.210 |
|  | College or above | 2.44 | 0.52 | 0.23-1.18 | 0.118 | 0.47 | 0.71 | 0.26-1.90 | 0.495 |
| Marital status |  | 9.23 |  |  | **0.010** | 3.56 |  |  | 0.169 |
|  | Never married | - | 1.00 | - | - | - | 1.00 | - | - |
|  | Married | 8.44 | 0.25 | 0.10-0.64 | **0.004** | 2.74 | 0.34 | 0.09-1.22 | 0.098 |
|  | Divorced or Widowed | 0.14 | 0.70 | 0.11-4.42 | 0.704 | 0.00 | 0.99 | 0.10-9.70 | 0.998 |
| The type of school |  |  |  |  |  |  |  |  |  |
|  | Private school | - | 1.00 | - | - | - | - | - | - |
|  | Public school | 0.38 | 0.78 | 0.35-1.73 | 0.537 | - | - | - | - |
| Income satisfaction |  |  |  |  |  |  |  |  |  |
|  | No | - | 1.00 | - | - | - | 1.00 | - |  |
|  | Yes | 8.45 | 0.35 | 0.17-0.71 | **0.004** | 4.02 | 0.40 | 0.16-0.98 | **0.045** |
| BMI |  |  |  |  |  |  |  |  |  |
|  | Normal weight | - | 1.00 | - | - | - | - | - | - |
|  | Underweight /Overweight /Obesity | 0.38 | 0.80 | 0.39-1.64 | 0.537 | - | - | - | - |
| Depression |  |  |  |  |  |  |  |  |  |
|  | No | - | 1.00 | - | - | - | 1.00 | - | - |
|  | Yes | 18.52 | 5.22 | 2.46-11.06 | **<0.001** | 3.11 | 2.32 | 0.91-5.92 | 0.078 |
| Age(years) |  | 8.12 | 0.94 | 0.91-0.98 | **0.004** | 0.04 | 1.01 | 0.93-1.09 | 0.834 |
| Years of teaching |  | 6.30 | 0.96 | 0.93-0.99 | **0.012** | 0.07 | 1.01 | 0.95-1.07 | 0.788 |
| Perceived stress |  | 21.28 | 1.15 | 1.08-1.22 | **<0.001** | 9.56 | 1.11 | 1.04-1.19 | **0.002** |

^#^ This multivariate factor regression analysis was based on n=134 and adjustment for all variables with the exception of the type of school and BMI;

Boldface indicates significant at *P*<0.05; BMI: body mass index.
